# Age disparities in adverse reactions of drugs used in pain therapies in Switzerland

**DOI:** 10.1038/s41598-025-28959-7

**Published:** 2025-11-25

**Authors:** Lucia Gasparovic, Andrea Michelle Burden, Oliver Senn, Stefan Markun, Boris B. Quednow, Stefan Neuner-Jehle, Thomas Stammschulte, Stefan Weiler

**Affiliations:** 1 Institute of Pharmaceutical Sciences, ETH Zurich, Zurich, Switzerland; 2 Institute of Primary Care, University of Zurich, University Hospital Zurich, Pestalozzistrasse 24, CH-8091 Zurich, Switzerland; 3 Experimental and Pharmacopsychology and Psychological Addiction Research, Department of Adult Psychiatry and Psychotherapy, University Hospital of Psychiatry Zurich, University of Zurich, Zurich, Switzerland; 4 Pharmacovigilance, Safety of Medicines Division, Swissmedic, Swiss Agency for Therapeutic Products, Bern, Switzerland

**Keywords:** Diseases, Drug discovery, Health care, Medical research

## Abstract

Pain therapies are prescribed to a significant proportion of the population but also associated with adverse drug reactions (ADRs). The prescription of pain therapies requires careful consideration of their safety profiles in the context of patient-specific characteristics, such as age-specific vulnerabilities, which are, however, still understudied so far. The aim of this study was to describe ADRs reported for drugs used in pain therapies and investigate age disparities. We conducted a descriptive comparative analysis of individual case safety reports (ICSRs) of drugs used in pain therapies from the global ADR database VigiBase between September 29, 1991, and December 31, 2022, from Switzerland. Comparisons were drawn between younger and older adults (18–74 years vs. 75 + years). Disproportionality (reporting odds ratio, ROR) of serious ADRs was assessed between age groups. A total of 17,228 ICSRs were analysed (58% female, 24% age 75 +). Across both age groups, the most frequently reported ADRs were related to the nervous system (23%), gastrointestinal system (20%), and general health and administration site conditions (20%). Serious ADRs were more common in the older population compared to younger adults (69% vs. 54%) with an ROR of 1.9. Fatal ADRs were also disproportionally higher in older adults (ROR 1.9). Hemorrhage was the most frequent fatal reaction. Commonly used pain therapies can lead to ADRs with a pronounced impact, especially in older adults. A deeper understanding of the safety profiles of these drugs should aid healthcare professionals in making more informed, safer treatment decisions.

**Supplementary Information:**

The online version contains supplementary material available at 10.1038/s41598-025-28959-7.

## Introduction

Pain medication involves a variety of different pharmacological classes potentially added to non-pharmacological measures. Common pain medications include non-steroidal anti-inflammatory drugs (NSAIDs) and opioids for a variety of pain types, often complemented by adjuvant pain medication, antimigraine drugs or other specific products containing paracetamol and metamizole. Adverse drug reactions (ADRs) to pain medications range from common – usually less severe effects – to infrequent potentially serious outcomes. The potential of harm was recently elucidated especially for opioids in Switzerland^1,2^. However, non-opioids also pose serious and potentially life-threatening risks, including cardiovascular events, hepatic and renal disorders, gastrointestinal perforation, or blood disorders, such as agranulocytosis.

Pharmacological pain treatment considers factors such as the type and degree of pain, patient characteristics, comorbidities, concomitant medications, and patient preferences. The decision for a drug class and specific drugs within the classes involves an individual benefit-risk analysis to balance analgesic efficacy against potential adverse reactions and harms. This requires a deep and nuanced understanding of the safety profiles of pain medications in the context of patient-specific characteristics, particularly when treating populations who are more vulnerable to ADRs, such as older adults.

Pain medication is widely prescribed in Switzerland, with recent reports indicating an increase in their usage^3–5^. Even among young adults, at 20 years old, the prevalence of paracetamol use was at 23%^6^ and opioid analgesics had been used by 2.6%^7^.

This nationwide analysis aimed to elucidate ADRs associated with pain therapies in Switzerland and compared the seriousness and reaction types occurring in younger and older adults. By examining a comprehensive set of spontaneous reports of ADRs, this study aims to describe specific reactions of Swiss pain therapies and to identify potential age-related disparities in ADR distribution using Reporting Odds Ratios (RORs).

## Methods

### Study design

This is a descriptive analysis of pharmacovigilance data. We analysed individual case safety reports (ICSRs) of ADRs of pain medications reported in Switzerland between September 29, 1991, and December 31, 2022. Disproportionality between the reporting of serious ADRs in younger vs older adults was assessed using RORs.

### Data source and variables

This study used data from the WHO pharmacovigilance database VigiBase^8^. VigiBase is a global database of ADRs. ADRs are reported spontaneously to national pharmacovigilance centres or drug manufacturers by healthcare professionals and non-healthcare professionals. The ADRs are then recorded as ICSRs including patient characteristics, the suspect drug and the experienced reaction. Medications in WHODrug are classified using the ATC system and clustered into Standardised Drug Groupings (SDG), to allow for grouping of medications with one or more properties in common, based on chemical structure, pharmacological effect or metabolic pathway^9^. Reactions are reported as MedDRA preferred terms (PTs). PTs are distinct descriptors for symptoms, diagnoses, or investigations. All PTs are categorised into overarching MedDRA System Organ Classes (SOC), which group the PTs by aetiology, manifestation site or purpose^10^. Each ICSR can report on multiple drugs and reactions and can therefore be categorised into multiple SDGs and SOCs.

### Selection of ICSRs

We included all reports from Switzerland between September 29, 1991 (date of the first report on drugs used in pain therapies in Vigibase), and December 31, 2022. All reports had to include at least one suspect drug used in pain therapies in accordance with the WHODrug^11^ SDG^12^. This SDG contains the subgroups *adjuvant pain medications*, *analgesia producing opioids*, *antimigraine preparations*, *nonsteroidal anti-inflammatory drugs used in pain therapies*, and *other analgesic drugs used in pain therapies*. A complete list of the substances included in the SDG is not publicly available. Reports with missing age or sex, age under 18 years, and pregnancy cases were excluded. Duplicate reports were identified using the vigiMatch™ algorithm, a statistical method for identifying multiple reports of the same case^13^.

### Statistical analysis

ICSRs were analysed descriptively. ICSRs were stratified according to individuals’ age into “older adults” (75 years and older), and “younger adults” (18 to 74 years). We summarized ICSR characteristics, including sex, reporter, seriousness and seriousness criteria, and fatality. Seriousness and fatality were determined using the standardized ratings provided by VigiBase, which are assigned by the Uppsala Monitoring Centre according to ICH E2A guidelines^14^. This ensured that all reactions meeting serious or fatal criteria were captured in the analysis. Categorical variables were described in absolute numbers and percentage of all ICSRs. Continuous variables (age) were described with a mean and standard deviation, and a median and minimum and maximum value.

To determine the most relevant drugs and reactions, in each SDG subgroup, the top drugs for most frequently reported “suspect” drugs for the ADRs were analysed by age group: This included the 10 most frequently reported drugs for the subgroups of “analgesia producing opioids”, and “NSAIDs”, “adjuvant pain medications”, “antimigraine preparations”, for substances used in pain therapies.

The reported reactions of the top reported drugs per subgroup were examined based on the MedDRA PTs. To optimize the drug-reaction pairs, the MedDRA PTs were assigned a MedDRA Higher Level Term (HLT). In MedDRA each PT can be linked to multiple HLTs (1 to n). For this analysis, we assigned a single HLT for each preferred term (1 to 1) as follows: (1) The frequency for each HLT occurrence was calculated across the MedDRA database. (2) For PTs associated with multiple HLTs, the HLT with the highest occurrence was selected, and the others were discarded. The steps were repeated three times, ensuring that each PT was consistently associated with HLTs of equal frequency. In cases where multiple HLTs remained, the first HLT in alphabetical order was chosen to establish a one-to-one PT-HLT relationship. The procedure is visualized in Supplement Fig. 1.

The drug-reaction pairs were quantified for selected drugs in specific SDG, focusing on drugs frequently reported and those particularly relevant to Switzerland based on clinical experience. For the SDG “NSAIDs” the substances diclofenac, ibuprofen, mefenamic acid, and metamizole were selected. For “analgesia producing opioids” we chose morphine, oxycodone, and tramadol and for “other analgesia producing drugs” the reactions of gabapentin, pregabalin, and paracetamol were analysed.

The ROR for seriousness and seriousness criteria in older vs. younger adults was calculated as *ROR* = *(n*_older, serious_*n_*younger, non-serious*_*)/(n*_younger, serious_*n_*older, non-serious*_*)* with the respective confidence interval. The formula is visualized in the Supplement.

All analyses were conducted with R^15^ (version 4.2.3) and RStudio (version 2022.07.2). The flowchart was created with the R package *flowchart*^16^. The manuscript was prepared in accordance with the STROBE guideline and the READUS-PV guideline and checklist^17,18^.

### Meeting presentations

This study was presented at the Swiss Society of General Internal Medicine’s Spring Congress; May 29–31, 2024; Basel, Switzerland; and at the International Society for Pharmacoepidemiology’s Annual Meeting; August 24–28, 2024; Berlin, Germany.

## Results

### Report characteristics

**Fig. 1:**
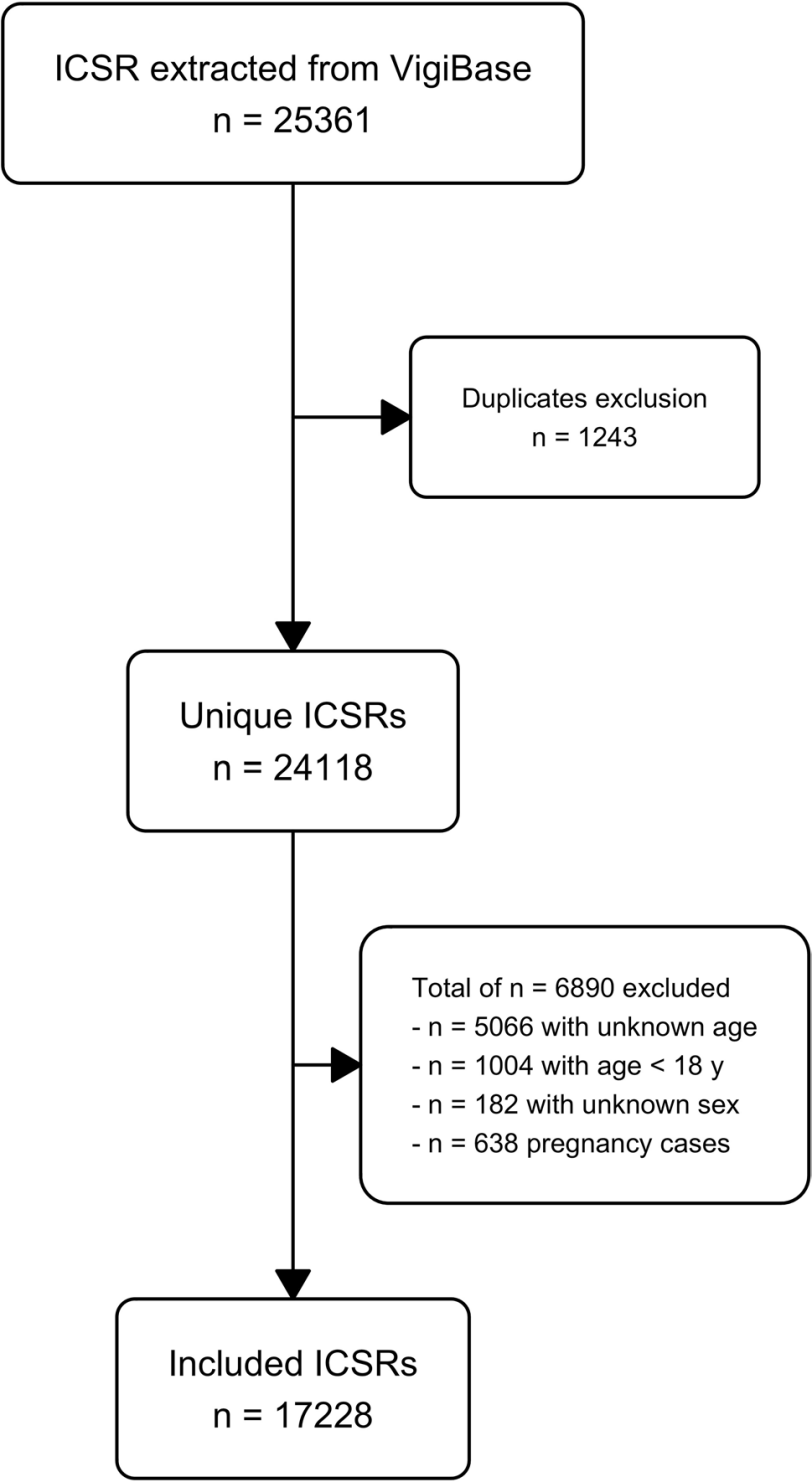
Flowchart of individual case safety report (ICSR) inclusion and exclusion process.

After removing duplicates (n = 1,243), cases with unknown age (n = 5,066) or sex (n = 182), age < 18 (n = 1,004), and pregnancy cases (n = 638), a total of 17,228 ICSRs from Switzerland with pain medications were analysed (Fig. 1).

Table 1 summarizes the report characteristics. Overall, 58.4% were female, mean age was 58 years and 23.5% were 75 years or older. Of the 17,228 ICSRs, 4,731 (27.5%) involved on at least one SDG adjuvant pain medication, 3,919 (22.7%) included an antimigraine preparation, 5,784 (33.6%) reported an NSAID, 2,123 (12.3%) included an analgesic opioid, and 3,013 (17.5%) involved at least one drug from the SDG of “other drugs used in pain therapies”.

**Table 1:** Characteristics of the individual case safety reports (ICSRs) of drugs used in pain therapies stratified by age.

	All adults	Younger adults (18–74 years)	Older adults (75 + years)
	Counts, No. (%)	Counts, No. (%)	Counts, No. (%)
ICSRs	17,228 (100.0)	13,183 (76.5)	4045 (23.5)
Sex			
Male	7172 (41.6)	5608 (42.5)	1564 (38.7)
Female	10,056 (58.4)	7575 (57.5)	2481 (61.3)
Age			
Mean (SD)	58.2 (18.9)	50.8 (15.1)	82.0 (5.26)
Median [Min, Max]	59.0 [18.0, 113]	52.0 [18.0, 74.0]	81.0 [75.0, 113]
Serious			
Yes	9939 (57.7)	7145 (54.2)	2794 (69.1)
No	3673 (21.3)	3098 (23.5)	575 (14.2)
Missing	3616 (21.0)	2940 (22.3)	676 (16.7)
Fatal			
Yes	913 (5.3)	539 (4.1)	374 (9.2)
No	16,315 (94.7)	12,644 (95.9)	3671 (90.8)
Drug category			
Adjuvant pain medications	4731 (27.5)	3879 (29.4)	852 (21.1)
Antimigraine preparations	3919 (22.7)	3161 (24.0)	758 (18.7)
NSAIDs	5784 (33.6)	3917 (29.7)	1867 (46.2)
Opioids	2123 (12.3)	1595 (12.1)	528 (13.1)
Other drugs used in pain therapies	3013 (17.5)	2397 (18.2)	616 (15.2)
Number of reported drugs			
Median [Min, Max)	3 [1, 42]	3 [1, 42]	5 [1, 35]
1	4290 (24.9)	3711 (28.1)	579 (14.3)
2	2718 (15.8)	2298 (17.4)	420 (10.4)
3	2010 (11.7)	1683 (12.8)	327 (8.1)
4 +	8210 (47.6)	5491 (41.7)	2719 (67.2)

*SD* standard deviation.

### System organ classes (SOC)

Overall, the three most frequently affected SOCs were nervous system disorders (23.4%), gastrointestinal disorders (19.7%), and general disorders and administration site conditions (19.5%) with at least one reaction in the respective SOC (Supplement Table 3). In older adults, gastrointestinal disorders (23.4%), nervous system disorders (21.9%), and blood and lymphatic system disorders (16.2%) were most common. In younger adults, nervous system disorders (23.9%), general disorders and administration site conditions (20.6%), and gastrointestinal disorders (18.3%) were most frequent (see Supplement Table 3).

**Fig. 2:**
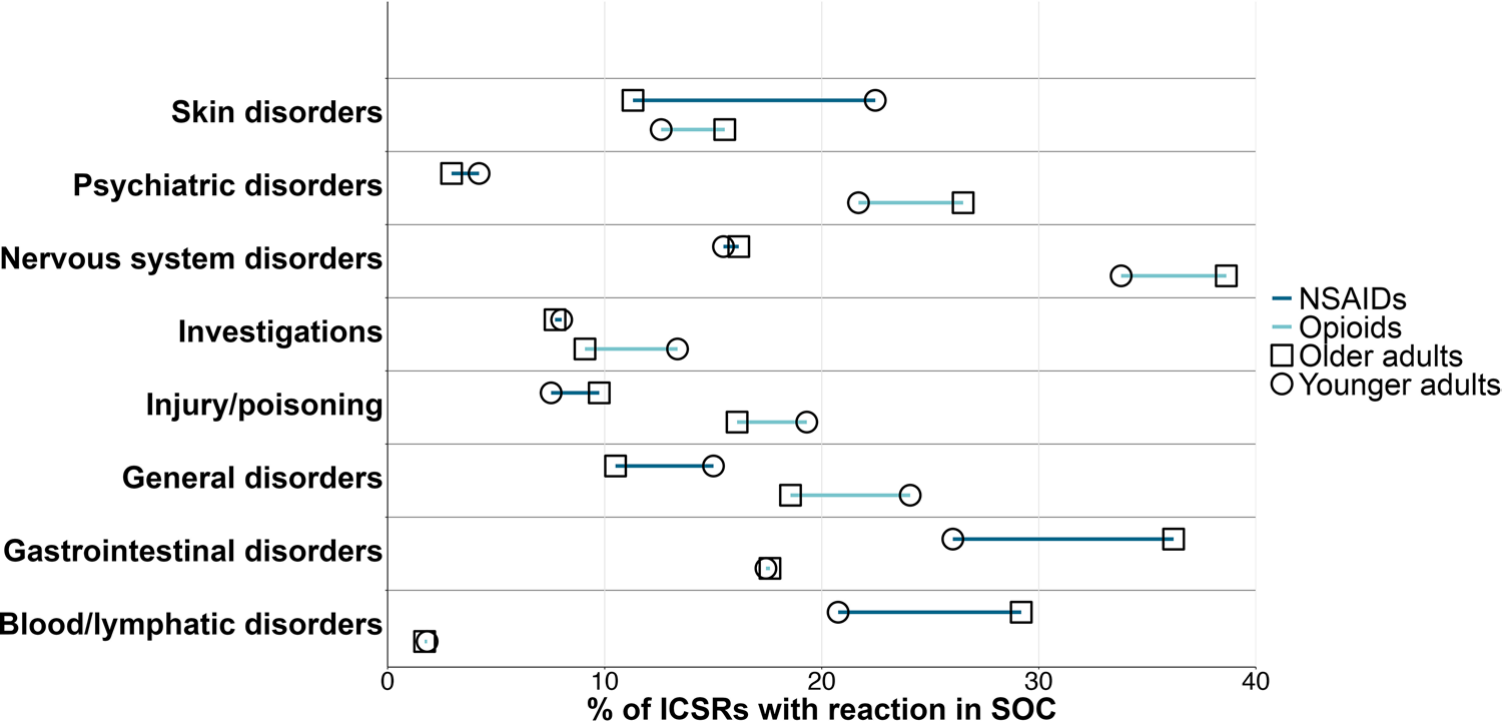
Most affected system organ classes (SOCs) and percent of individual case safety reports (ICSRs) in younger and older adults with one or more reaction in the respective SOC for the drug classes NSAIDs and opioids.

Figure 2 illustrates the top 8 most affected SOCs, stratified by age group and drug classes of NSAIDs and opioids, as clearly defined subgroups within the SDG category. For opioids, nervous system disorders (35.0%) were the most reported adverse reactions, while for NSAIDs gastrointestinal (29.3%) and blood and lymphatic disorders (23.5%) were predominant.

### Drugs and reactions

The most frequently reported suspect drugs overall were acetylsalicylic acid, paracetamol, venlafaxine, diclofenac, and metamizole. The top reported drugs for each SDG subgroup are detailed in Supplement Table 2.

Table 2 presents drug-reaction pairs, i.e., the top reported drugs per SDG subcategory associated with the reactions coded as MedDRA HLTs, stratified by age group. The MedDRA PTs categorized in each HLT are listed in Supplement Table 4.

**Table 2:** Most frequently reported reactions (Higher Level Terms, HLTs) for selected top reported drugs from the SDG subgroups NSAIDs used in pain therapies, analgesia producing opioids, and other drugs used in pain therapies stratified by age group.

Younger adults (18–74 years)		Older adults (75 + years)		
HLT	Counts, No. (%)	HLT		Counts, No. (%)
NSAIDs used in pain therapies				
Diclofenac				
Total reactions	1535 (100.0)	Total reactions		595 (100.0)
Renal failure and impairment	68 (4.4)	Non-site specific gastrointestinal haemorrhages		98 (16.5)
Non-site specific gastrointestinal haemorrhages	57 (3.7)	Renal failure and impairment		48 (8.1)
Nausea and vomiting symptoms	51 (3.3)	Gastric ulcers and perforation		37 (6.2)
Rashes, eruptions and exanthems NEC	48 (3.1)	Anaemias NEC		36 (6.1)
Allergic conditions NEC	41 (2.7)	Haemorrhages NEC		29 (4.9)
Ibuprofen				
Total reactions	1607 (100.0)	Total reactions		291 (100.0)
Renal failure and impairment	77 (4.8)	Non-site specific gastrointestinal haemorrhages		45 (15.5)
Hepatocellular damage and hepatitis NEC	59 (3.7)	Renal failure and impairment		18 (6.2)
Rashes, eruptions and exanthems NEC	57 (3.5)	Gastric ulcers and perforation		15 (5.2)
Neutropenias	51 (3.2)	Anaemias NEC		13 (4.5)
Non-site specific gastrointestinal haemorrhages	49 (3.0)	Haemorrhages NEC		9 (3.1)
Mefenamic acid				
Total reactions	830 (100.0)	Total reactions		196 (100.0)
Renal failure and impairment	43 (5.2)	Non-site specific gastrointestinal haemorrhages		23 (11.7)
Nausea and vomiting symptoms	42 (5.1)	Renal failure and impairment		18 (9.2)
Allergic conditions NEC / Rashes, eruptions and exanthems NEC	29 (3.5) 29 (3.5)	Rashes, eruptions and exanthems NEC		9 (4.6)
Diarrhoea (excl infective)	25 (3.0)	Anaemias NEC / Gastric ulcers and perforation		8 (4.1) 8 (4.1)
Non-site specific gastrointestinal haemorrhages	22 (2.7)	Nausea and vomiting symptoms		7 (3.6)
Metamizole				
Total reactions	1485 (100.0)	Total reactions		396 (100.0)
Neutropenias	245 (16.5)	Neutropenias		64 (16.2)
Rashes, eruptions and exanthems NEC	61 (4.1)	Marrow depression and hypoplastic anaemias		23 (5.8)
Marrow depression and hypoplastic anaemias	50 (3.4)	Leukopenias NEC / Thrombocytopenias		19 (4.8) 19 (4.8)
Leukopenias NEC	39 (2.6)	Sepsis, bacteraemia, viraemia and fungaemia NEC		15 (3.8)
Febrile disorders	35 (2.4)	Anaemias NEC / Non-site specific gastrointestinal haemorrhages		12 (3.0) 12 (3.0)
Analgesia producing opioids				
Morphine				
Total reactions	705 (100.0)	Total reactions		239 (100.0)
Cortical dysfunction NEC / Dyssomnias	33 (4.7) 33 (4.7)	Cortical dysfunction NEC		21 (8.8)
Neurological signs and symptoms NEC	27 (3.8)	Rashes, eruptions and exanthems NEC		15 (6.3)
Nausea and vomiting symptoms	26 (3.7)	Neurological signs and symptoms NEC		10 (4.2)
Overdoses NEC	20 (2.8)	Deliria / Mental disorders NEC / Muscle tone abnormal / Nausea and vomiting symptoms / Overdoses NEC		8 (3.3) 8 (3.3) 8 (3.3) 8 (3.3) 8 (3.3)
Product administration errors and issues	19 (2.7)	Coma states / Disturbances in consciousness NEC		7 (2.9) 7 (2.9)
Oxycodone				
Total reactions	396 (100.0)	Total reactions		207 (100.0)
Nausea and vomiting symptoms	25 (6.3)	Cortical dysfunction NEC		11 (5.3)
Substance related and addictive disorders	16 (4.0)	Dyssomnias		10 (4.8)
Dyssomnias / Gastrointestinal atonic and hypomotility disorders NEC	12 (3.0) 12 (3.0)	Neurological signs and symptoms NEC		9 (4.3)
Cortical dysfunction NEC / Hepatobiliary function diagnostic procedures / Neurological signs and symptoms NEC	11 (2.8) 11 (2.8) 11 (2.8)	Anxiety symptoms / Hallucinations (ecxl sleep-related)		7 (3.4) 7 (3.4)
Interactions / Product administration errors and issues	9 (2.3) 9 (2.3)	Gastrointestinal atonic and hypomotility disorders NEC / Nausea and vomiting symptoms / Non-site specific injuries NEC		6 (2.9) 6 (2.9)
Tramadol				
Total reactions	981 (100.0)	Total reactions		322 (100.0)
Nausea and vomiting symptoms	75 (7.6)	Cortical dysfunction NEC		23 (7.1)
Cortical dysfunction NEC / Neurological signs and symptoms NEC	42 (4.3) 42 (4.3)	Muscle tone abnormal		18 (5.6)
Overdoses NEC	30 (3.1)	Neurological signs and symptoms NEC		16 (5.0)
Muscle tone abnormal	29 (3.0)	Nausea and vomiting symptoms		13 (4.0)
Dyssomnias	28 (2.9)	Asthenic conditions		12 (3.7)
Other analgesic drugs used in pain therapies^a^				
Gabapentin				
Total reactions	338 (100.0)	Total reactions		135 (100.0)
Neurological signs and symptoms NEC	16 (4.7)	Off label uses		12 (8.9)
Nausea and vomiting symptoms	10 (3.0)	Dyssomnias		9 (6.7)
Cortical dysfunction NEC / Rashes, eruptions and exanthems NEC	9 (2.7) 9 (2.7)	Cortical dysfunction NEC		8 (5.9)
Anxiety symptoms / Asthenic conditions	8 (2.4) 8 (2.4)	Dyskinesias and movement disorders NEC / Neurological signs and symptoms NEC		7 (5.2) 7 (5.2)
Dyssomnias / Headaches NEC	7 (2.1) 7 (2.1)	General signs and symptoms NEC / Non-site specific injuries NEC		6 (4.4) 6 (4.4)
Paracetamol				
Total reactions	2694 (100.0)	Total reactions		660 (100.0)
Hepatocellular damage and hepatitis NEC	211 (7.8)	Hepatocellular damage and hepatitis NEC		65 (9.8)
Poisoning and toxicity	203 (7.5)	Hepatobiliary function diagnostic procedures		36 (5.5)
Nausea and vomiting symptoms	167 (6.2)	Rashes, eruptions and exanthems NEC		23 (3.5)
Hepatobiliary function diagnostic procedures	153 (5.7)	Coagulation and bleeding analyses		20 (3.0)
Overdoses NEC	81 (3.0)	Nausea and vomiting symptoms / Renal failure and impairment		16 (2.4) 16 (2.4)
Pregabalin				
Total reactions	1121 (100.0)	Total reactions		356 (100.0)
Neurological signs and symptoms NEC	40 (3.6)	Cortical dysfunction NEC		14 (3.9)
Dyssomnias	34 (3.0)	Non-site specific injuries NEC		12 (3.4)
Therapeutic and nontherapeutic responses	28 (2.5)	Neurological signs and symptoms NEC / Rashes, eruptions and exanthems NEC		11 (3.1) 11 (3.1)
Asthenic conditions	26 (2.3)	General signs and symptoms NEC / Therapeutic and nontherapeutic responses		10 (2.8) 10 (2.8)
Cortical dysfunction NEC	25 (2.2)	Dyssomnias / Gait disturbances		8 (2.2) 8 (2.2)

Reactions are listed as percent of total reactions for each respective drug within each age category. Where multiple reactions had the same frequency, they are reported in the same cell and separated by “/”. Percentages reflect the proportion of all reactions for each drug within the age category with the total reactions (100%) shown under “Total reactions”. Number/percentage of ICSRs with reaction shown for each drug do not add up to total number of reactions/100% for the respective drug, as only the five most frequent reactions are shown.

*NEC* not elsewhere classified.

^a^ Due to a low number of reports only the four most frequently reported drugs in the subgroup “other drugs used in pain therapies” were analysed.

In younger and older adults, renal failure and impairments were frequently reported with NSAIDs including diclofenac, ibuprofen, and mefenamic acid. In younger adults, allergic conditions, and dermatologic reactions (rashes, eruptions and exanthems) were also common. For older adults, gastrointestinal haemorrhages were the most frequently reported reactions with these drugs. For metamizole, the most common reactions in both age groups were neutropenias, and other cytopenias and anaemias.

For analgesic opioids, such as morphine, oxycodone and tramadol, cortical dysfunctions – such as confusional state, agnosia, aphasia, and apraxia – were among the most frequently reported reactions in both age categories ranging from 2.8 to 8.8% by substance and age (Table 2). Neurological symptoms, including dizziness, respiratory depression, and disorientation, were also common, accounting for 2.8 to 5.0% of all reactions. Other frequently reported reactions included nausea and vomiting, especially in younger adults, as well as dyssomnias. Overdoses (intentional, prescribed, or not specified) were frequently reported in younger adults for morphine (2.8%) and tramadol (3.1%).

For gabapentinoids (gabapentin and pregabalin), frequently reported reactions were primarily neurological, including dyssomnias (2.2–6.7%), neurological signs and symptoms (3.1–5.2%), and cortical dysfunctions (2.2–5.9%). In older adults, “off label use” was the most commonly reported term associated with gabapentin (8.9%). For paracetamol, hepatocellular damage and hepatitis were the most frequently reported reactions in both age groups, comprising 7.8% of reactions in younger and 9.8% in older adults. Among older adults, renal failure and impairment were also frequently reported (2.4%), while in younger adults, poisoning and toxicity (7.5%) and overdoses (3.0%) were prominent with paracetamol.

### Serious reactions and death

Overall, 57.7% of ICSRs were classified as serious, with a higher incidence in older adults (69.1%) than in younger adults (54.2%). Hospitalization was the most frequently reported seriousness criterion, occurring in 34.6% of all ICSRs (see Table 3).

**Table 3:** Seriousness and seriousness criteria of ICSRs of drugs used in pain therapies. Note: A low ROR does not indicate a protective effect but suggests that the drug-event combination is not reported disproportionately in the database.

	Younger adults (18–74 years)	Older adults (75 + years)	Older vs. younger adults
	Counts, No. (%)	Counts, No. (%)	ROR (95% CI)
ICSRs	13,183 (100.0)	4045 (100.0)	
Serious			
Yes	7145 (54.2)	2794 (69.1)	1.9 (1.8–2.0)*
Seriousness criteria			
Hospitalization	3989 (30.3)	1978 (48.9)	1.9 (1.7–2.1)*
Congenital	0 (0.0)	1 (0.0)	NA
Lifethreatening	655 (5.0)	164 (4.1)	0.6 (0.5–0.7)*
Death	371 (2.8)	266 (6.6)	1.9 (1.6–2.2)
Disabling	234 (1.8)	71 (1.8)	0.8 (0.6–1.0)
Other	2917 (22.1)	680 (16.8)	0.5 (0.5–0.6)*
Missing	37 (0.3)	12 (0.3)	0.8 (0.4–1.5)

* p < 0.05.

Serious reactions were reported more frequently in older compared to younger adults, with an ROR of 1.9 (95% CI 1.8–2.0). Similarly, serious reactions resulting in death were more common in older adults compared to younger adults (ROR 1.9; 95% CI 1.6–2.2), as shown in Table 3.

To assess the robustness of our age-related findings, we conducted a post-hoc sensitivity analysis by recalculating the RORs using an alternative age cut-off of 65 years; the resulting RORs showed only minimal deviations from the original stratification, confirming that the observed age-related differences in adverse drug reaction reporting were consistent across both age group definitions (see Supplement Table 5).

The reports for medications and ADRs most frequently associated with death are listed in Supplement Table 6. Acetylsalicylic acid was the most frequently reported suspect drug cases resulting in death, accounting for 33.5% of reports in older and 15.4% in younger adults. The most frequently associated reaction was hemorrhage. Additional drugs and reactions frequently reported in fatal serious cases are detailed in Supplement Table 6.

## Discussion

This study provides the first comprehensive analysis of ICSRs of drugs used in pain therapies in Switzerland. Our analysis of 17,228 ICSRs revealed notable age-related differences in the nature and severity of reported reactions to drugs commonly used in pain therapy. Serious reactions were reported twice as often in older adults based on the ROR, with a higher incidence of serious adverse reactions leading to death and hospitalisations compared to younger adults.

The higher frequency of serious adverse events and hospitalisations in older adults is consistent with previous studies^19,20^. A systematic review by Bouvy et al. found that 5.8 to 28.2% of hospital admissions in older adults were due to ADRs (used not only for pain treatment), compared to 0.8 to 12.8% in the general adult population^19^. Similarly, in a surveillance study Budnitz et al. reported that older adults (65 years and older) were 2.4 times more likely to experience adverse drug events and 6.8 times more likely to require hospitalization^20^. Other studies have identified that the drug classes frequently involved in adverse reactions leading to hospitalisation include antithrombotic agents, diuretics, and antimicrobial agents as well as NSAIDs and central nervous system agents, such as opioid analgesics^20–23^. These findings underscore the heightened vulnerability of older adults, who often experience polypharmacy and frailty and are exposed to high risk drug classes^24^. In the present study, the median number of reported drugs per patient was higher in older adults (median 5 [range 1–35]) compared with younger adults (18–74 years; median 3 [range 1–42]). A larger proportion of older adults were reported with four or more medications (67.2% vs. 41.7%), highlighting the greater prevalence of polypharmacy in this group.

In our data, acetylsalicylic acid, ibuprofen, diclofenac, and metamizole were among the most frequently reported drugs, with acetylsalicylic acid being the leading suspect in serious fatal cases, reflecting its bleeding risks as an antithrombotic agent. While physicians might be reluctant to prescribe NSAIDs due to their poor risk–benefit balance in older adults^25–27^, they are often acquired over-the-counter (OTC). Sawyer et al. reported that 41% of community-dwelling older adults used OTC NSAIDs for pain relief^28^. However, Schmiedl et al. reported that only a small proportion (around 7%) of preventable ADRs leading to hospitalization were related to self-medication, with such cases occurring even less frequently among older adults, suggesting that OTC drugs contribute relatively little to serious, hospitalization-requiring ADRs^29^. While metamizole and paracetamol are recommended alternatives^26,27^, they were also frequently implicated in serious fatal reactions in our study. Metamizole has been withdrawn from the market in several countries, such as United Kingdom, France, Sweden, Norway, the United States, Canada, and Australia due to risks of agranulocytosis, and hepatotoxicity^30,31^. It is, however, frequently used in Switzerland where its use has further increased by 84% from 2014 to 2019^32,33^. The European Medicines Agency and Swissmedic have recently issued recommendations to mitigate the risk of agranulocytosis through early detection. Metamizole product leaflets will subsequently be updated in the EU as well as in Switzerland^34,35^. Paracetamol-related fatalities, primarily due to overdose and liver injury, were noted in both younger and older adults, with additional risk of bone marrow depression and hypoplastic anaemias in older adults. Although paracetamol-induced liver injury is well-documented, little is known about factors that exacerbate this risk^36,37^. However, our group has linked increasing poisonings to the introduction of 1000 mg paracetamol tablets in Switzerland^38^. Lowering prescribed doses of paracetamol could reduce hepatotoxicity risks, while bone marrow depression and cytopenia remain rare but known side effect of paracetamol^39^.

For analgesic opioids, neurological symptoms were the most common reported adverse reactions in both younger and older adults. Younger adults reported more gastrointestinal symptoms, such as nausea and vomiting, and had higher rates of overdoses and substance use disorders, particularly with oxycodone. Notably, no differences were observed in the adverse reactions between the weak opioid tramadol and the stronger opioids like oxycodone and morphine. These findings align with pharmacovigilance reports from other countries like the Netherlands^40^ and France^41^. Particularly, central nervous system effects, such as dizziness or somnolence, are particularly concerning in older adults, as they increase the risk of falls, injuries, and fractures^42,43^.

For gabapentin, the most frequently reported ADR in younger adults was off-label use. While approved in Switzerland for the treatment of epileptic seizures and neuropathic pain, gabapentin is used off-label for conditions like restless leg syndrome, hot flushes in perimenopause, and spasticity in patients with multiple sclerosis^44^. Additionally, gabapentinoids are sometimes prescribed for chronic nociceptive pain to reduce reliance on NSAIDs or opioids. Between 2013 and 2018, gabapentinoid claims in Switzerland increased by 46%^45^. However, these medications, especially pregabalin, carry a risk of non-medical use for recreational purposes, with reports of poisoning and non-medical use rising between 2010 and 2020^46,47^. Non-medical use seems especially prevalent and increasing in refugees, migrants, and incarcerated people, both in Switzerland and elsewhere^48–51^. In our study, central nervous ADRs such as cortical dysfunction, neurological signs and symptoms were most frequently associated with the use of gabapentinoids. As with opioid analgesics, these ADRs lead to an increased risk in secondary injuries, especially in older adults who combined opioids and gabapentinoids^52,53^.

This study has notable strengths, including its large dataset, encompassing 17,228 ICSRs and providing a comprehensive picture of ADR of drugs used in pain therapies across the country. Furthermore, by including a wide range of drugs used for pain management, both primary analgesic agents and also adjuvants, the study offers valuable insights into the safety profile of commonly prescribed drugs and therefore reflecting real-life use. Additionally, the real-life context of the data enhances its relevance, capturing a broad spectrum of patients’ characteristics and treatment practices. Distinguishing between older and younger adults provided important insights into a population that, while often in need of effective pain treatment, is more vulnerable to adverse outcomes.

The limitations of this study are inherent to the pharmacovigilance data set. Pharmacovigilance data relies on spontaneous reporting of adverse reactions, which means that an estimation of absolute incidences of adverse reactions is not possible. To account for this, we limited the analysis to descriptive and relative reporting patterns. All adverse events in this study were extracted from VigiBase, where they are coded using MedDRA terms. While the WHO definition of an ADR specifies reactions occurring at normal therapeutic doses for an indication, MedDRA terms also encompass events related to off-label use and overdoses. Consequently, our analysis reflects the database-reported terms without additional classification, and readers should interpret “ADRs” in this context as encompassing all spontaneously reported adverse drug events. We intentionally restricted our analysis to Swiss data to ensure methodological consistency, reporting comparability, and contextually appropriate interpretation within our national healthcare setting. This approach may limit the generalizability of the findings to other countries with different reporting systems and healthcare contexts. In our analysis we used RORs to calculate relative frequencies of reporting to be able to compare older and younger adults, assuming the age and the likelihood of reporting a reaction are independent. Nonetheless, RORs can merely provide a signal of a disproportionality and do not provide evidence of a causal harmful or protective effect. To investigate the differences between older and younger adults, this study distinguished two age categories, 18–74 and 75 years and older. However, future studies could investigate one or several more age categories to distinguish between young, middle aged and geriatric patients, as these could have systematic differences in both characteristics and treatment choices. The potential for underreporting of ADRs is a known issue of pharmacovigilance systems, which may result in an incomplete representation of the true incidence of adverse events. While the reporting of serious adverse reactions is mandatory under the Swiss Therapeutic Products Act^54^, non-serious reports are filed voluntarily and thus more rarely due to limited resources. Additionally, data on drug indications were often missing, leading to a lack of context of the drug’s use or the type of pain. This is particularly problematic for drugs with multiple indications, such as acetylsalicylic acid. However, we assumed that most adverse reactions, such as gastrointestinal ulcers and bleeding, would occur irrespective of the indication, although duration and dose might play an important role. Information on the duration and dosage of drug use, which might also vary by the indication, was frequently lacking, limiting the ability to assess the impact of these factors on adverse outcomes. The database did not contain information on whether the reported drugs were prescribed or purchased OTC, preventing us from distinguishing between these categories. This may influence ADR reporting patterns, particularly for medications available both by prescription and OTC. While the study provides nationwide data, it lacks detailed geographical information, which could offer insights into cantonal or language regional variations in prescribing and adverse event patterns. Similarly, given the study period spanning more than 30 years, it is likely that clinical guidelines, treatment restrictions, prescribing habits, and ADR reporting have evolved over time. While this does not impact the validity of the reported adverse reactions per se, it warrants caution when interpreting relative frequencies across drugs with differing or evolving patterns. Lastly, while this study highlights the vulnerability of older adults to serious ADRs, it does not account for other factors with a higher among older adults. Comorbidities and associated polypharmacy may have contributed to the observed differences in ADR profiles through increased susceptibility to drug–drug and drug–disease interactions, which could not be accounted for in this analysis.

In conclusion, this comprehensive study underscores the substantial risk of ADRs associated with commonly used pain medications, especially observed among older individuals compared to younger ones. In addition to age-related physiological changes, differences in comorbidity burden and polypharmacy play a significant role in shaping ADR risk profiles, underscoring the importance of individualized medication review and careful management of drug interactions in older patients. Reactions leading to hospitalisation or death in older adults were reported twice as frequently as in younger adults. The results of this real-world drug safety study emphasize the importance of individualized pain management, considering patients’ characteristics and substance risk profiles, while carefully balancing the benefit-risk profile of different medications to minimize adverse effects.

## Supplementary Information

Supplementary Information 1.

Supplementary Information 2.

## Data Availability

As data is licensed by the UMC it is not available for sharing. The code used for analysis can be shared upon reasonable request to Lucia Gasparovic ([lucia.gasparovic@pharma.ethz.ch] (mailto:lucia.gasparovic@pharma.ethz.ch)).
