# Supplementary material for: Age disparities in adverse reactions of drugs used in pain therapies in Switzerland
Source: Sci Rep. 2025 Nov 25;15:45301. doi: 10.1038/s41598-025-28959-7 (PMC12748878; doi:10.1038/s41598-025-28959-7)
Supplement: Supplementary file 1 — Supplementary Information 1. [file 41598_2025_28959_MOESM1_ESM.pdf]

The READUS-PV checklist

Section and topic	Item #	Checklist item	Location where item is reported
**Title**			
	1a	*If disproportionality analyses are a prominent component of the published study, the study should be identified as a "disproportionality analysis". The type of data and name of the database(s) should be specified.*	NA
	1b	*Report the name of adverse event(s) and/or drug(s) under study, when applicable.*	Title
**Introduction**			
Background	*2a*	*Describe the drug(s) and its utilization, the nature of the adverse event(s) under study and its frequency, and the existing knowledge on the drug-event combination.*	lines 60–67
	2b	*Specify the rationale for performing the analysis, e.g., as part of routine pharmacovigilance, to investigate an overall safety profile, or to assess a pre-specified hypothesis.*	80–84
	2c	*Explain why ICSR databases and disproportionality analysis are suitable to fill the knowledge gap.*	81–84
Objectives	*3*	*State specific objectives, identifying the adverse event(s), the drug(s), and the reference group, including any pre-specified hypothesis, if applicable.*	80–84
**Methods**			
Study design	*4a*	*Identify the study (i.e., "disproportionality analysis") and the type of data used (e.g., "individual case safety reports").*	88
	4b	*Provide an outline of the entire study design, including primary and sensitivity analyses performed, and other designs such as case-by-case analysis or literature review.*	88–90
Data description, access, and pre-processing	*5a*	*Specify the name of the database(s), the database(s) custodian, and the coverage. Specify the type/number of drugs included within the database and the thesaurus, taxonomies, or ontologies used for coding drugs and events.*	92–101
	5b	*Specify the extraction dates and describe and justify all choices used for data pre-processing, including any data transformation or exclusion, if appropriate.*	103–104, 109–110
Variables definition	*6a*	*Describe the study population, including any restriction.*	104–110
	6b	*Describe the nature and the meaning of key variables assessed in the work.*	94–100, 112–119
	6c	*Specify and justify any grouping of drugs or events. For drugs, specify and justify whether active ingredients/trade names/salts were considered and/or the selected role.*	95–100, 120–137
	6d	*Describe any additional data source used, the type of data, and how they interact with ICSRs.*	NA
Statistical methods	*7a*	*Present any descriptive analysis performed, specifying variables investigated, statistical tests, and significance thresholds.*	112–119
	7b	*Describe the measure(s) selected for the disproportionality analysis including any threshold used to identify signals of disproportionate reporting. Explain the reason for this choice if applicable.*	138–140
	7c	*Clearly describe any sensitivity analysis and any tool to control confounding, including any restriction, subgroup, stratification, adjustment, or interaction.*	229–233
	7d	*Specify the variables and methods used for the case-by-case analysis, including any algorithm or criteria used to assess causality, if performed.*	NA
	7e	*Specify any statistical methods used for other data sources.*	NA

**Results**			
Participants	*8a*	*Specify the number of individual case safety reports included at each stage, including reasons for exclusion.*	148–150, Fig 1
	8b	*Provide key demographic and clinical characteristics of cases, if possible comparing cases with any appropriate reference group.*	155–159, Table 1
Disproportionality analysis	*9*	*Present all results including confidence intervals. Present also results of sensitivity analyses, if performed.*	146–239
Case-by-case analysis	*10*	*Present the case-by-case analysis of key variables. Present the causality assessment, if applicable.*	NA
**Discussion**			
Key results	*11*	*Discuss key results with reference to study objectives and contextualize them within the current literature and other consulted sources. Clearly discriminate between expected reactions and emerging safety signals.*	243–302
External validity	*12a*	*Discuss the external validity of the results to the general population.*	304–311
	12b	*Discuss the potential relevance of results in clinical practice*	309–311, 353–361
	12c	*Propose further study designs if applicable*	328–330
Limitations	*13*	*Present general limitations, making clear that disproportionality analysis alone cannot prove causation or measure incidence, and specific limitations, including confounding and reporting bias and efforts to mitigate them.*	313–351
**Declarations**			
	14a	*Provide the source of funding/sponsorship and the role of the funders/sponsors for the present study and for any original study on which the present article is based.*	22–23, 374–375
	14b	*Clearly identify potential commercial and intellectual conflicts of interest (e.g., link to any drug/event investigated, whether financial, legal action, or software used).*	366–372
	14c	*Declare any institutional approval needed or granted in the investigation.*	382–389
	14d	*Include a statement on data availability, code availability (including the version of the statistical software used), and protocol registration.*	390–392
