# Supplementary material for: Age disparities in adverse reactions of drugs used in pain therapies in Switzerland
Source: Sci Rep. 2025 Nov 25;15:45301. doi: 10.1038/s41598-025-28959-7 (PMC12748878; doi:10.1038/s41598-025-28959-7)
Supplement: Supplementary file 2 — Supplementary Information 2. [file 41598_2025_28959_MOESM2_ESM.docx]

# Supplement

This Online Resource contains additional explanations to the methods of the study and additional tables and figures to supplement the results.

## Reduction procedure for MedDRA Higher Level Terms (HLTs)

**7**

**5**

**5**

**1**

| **PT** | **HLT** | **n** |  | **PT** | **HLT** | **n** |  | **PT** | **HLT** | **n** |  | **PT** | **HLT** | **n** |  | **PT** | **HLT** |
| --- | --- | --- | --- | --- | --- | --- | --- | --- | --- | --- | --- | --- | --- | --- | --- | --- | --- |
| a | A | 3 | **2** | a | A | 3 |  | a | A | 3 |  | a | A | 3 |  | a | A |
| a | B | 2 |  |  |  |  | **2** |  |  |  |  |  |  |  |  | b | A |
| b | A | 3 | **3** | b | A | 3 |  | b | A | 3 |  | b | A | 3 |  | c | A |
| b | C | 3 |  | b | C | 2 |  |  |  |  |  |  |  |  |  | d | D |
| c | A | 3 |  | c | A | 3 | **4** | c | A | 3 |  | c | A | 3 |  | e | C |
| c | B | 2 |  |  |  |  |  |  |  |  |  |  |  |  |  | f | E |
| c | C | 2 |  |  |  |  |  |  |  |  |  |  |  |  |  |  |  |
| d | D | 2 |  | d | D | 1 |  | d | D | 1 |  | d | D | 1 |  |  |  |
| e | C | 3 | **4** | e | C | 2 |  | e | C | 1 |  | e | C | 1 |  |  |  |
| e | D | 2 |  |  |  |  |  |  |  |  | **6** |  |  |  |  |  |  |
| f | E | 1 |  | f | E | 1 | **3** | f | E | 1 |  | f | E | 1 |  |  |  |
| f | F | 1 |  | f | F | 1 |  | f | F | 1 |  |  |  |  |  |  |  |

**Fig 1** Reduction procedure for Higher Level Terms (HLT)

1. The number of occurrences of each MedDRA Higher Level Term (HLT) as assigned to the MedDRA Preferred Terms (PT) is counted 🡪 n.
2. Where a PT (e.g., a) has multiple HLT, the one occurring more frequently (e.g., A) is selected for the next step.
3. Where a PT (e.g., b) has multiple HLT which all occur with the same frequency (e.g., A and C) all rows are retained for the next step.
4. Where a PT has only one HLT, this HLT is retained for the next step.
5. In the table with the rows retained in accordance with steps 2-4, the occurrences of the HLT are counted again. Steps 2-5 are repeated until no further rows can be reduced.
6. Where a PT (e.g., f) still has multiple HLT which occur with the same frequency (e.g., E and F), the HLT occurring first in the alphabet (E) is retained.
7. The final table has only one HLT assigned to each PT.

## Calculation of Reporting Odds Ratio and 95% confidence intervals

**Table 1** Contingency table for calculation of Reporting Odds Ratio (ROR)

|  | **Serious / fatal reactions** | **Non-serious / non-fatal reactions** | **Total** |
| --- | --- | --- | --- |
| Older adults | a | b | a + b |
| Younger adults | c | d | c + d |
| Total | a + c | b + d | a + b + c + d |

$$Reporting odds ratio \left( ROR \right)= \frac{a*d}{c*b}$$

$$95\% CI [lower, upper]= \left[ e^{\ln\left( ROR \right)-1.96*\sqrt{\frac{1}{a}+\frac{1}{b}+\frac{1}{c}+\frac{1}{d}}} , e^{\ln\left( ROR \right)+1.96*\sqrt{\frac{1}{a}+\frac{1}{b}+\frac{1}{c}+\frac{1}{d}}} \right]$$

**Table 2** Most frequently feported drugs in the Individual Case Safety Reports (ICSRs) of drugs used in pain therapies by Standardised Drug Grouping (SDG) subcategory, in descending order of frequency

| **NSAIDS used in pain therapies** | | **Antimigraine preparations** (cont.) | |
| --- | --- | --- | --- |
|  | Acetylsalicylic acid |  | Candesartan cilexetil |
|  | Diclofenac |  | Metoprolol |
|  | Metamizole |  | Amitriptyline |
|  | Ibuprofen |  | Atenolol |
|  | Mefenamic acid |  | Topiramate |
|  | Celecoxib |  | Verapamil |
|  | Rofecoxib |  | Botulinum toxin type a |
|  | Acemetacin |  | Propranolol |
|  | Nimesulide | **Adjuvant pain medications** | |
|  | Naproxen |  | Sertraline |
| **Analgesia producing opioids** | |  | Fluoxetine |
|  | Tramadol |  | Zoledronic acid |
|  | Morphine |  | Carbamazepine |
|  | Fentanyl |  | Lamotrigine |
|  | Methadone |  | Ibandronate |
|  | Oxycodone |  | Paroxetine |
|  | Buprenorphine |  | Bupropion |
|  | Codeine |  | Phenytoin |
|  | Tapentadol |  | Mianserin |
|  | Hydromorphone | **Other analgesic drugs used in pain therapies** | |
|  | Diamorphine |  | Paracetamol |
| **Antimigraine preparations** | |  | Pregabalin |
|  | Venlafaxine |  | Duloxetine |
|  | Valproate |  | Gabapentin |

## System Organ Class (SOC) of adverse event reports of drugs used in pain therapies

**Table 3** Number of cases with at least one adverse drug event in the listed System Organ Classes (SOCs)

| **System Organ Class (SOC)** | **All adults** | | **Younger adults**  (18-74 years) | | **Older adults**  (75+ years) | |
| --- | --- | --- | --- | --- | --- | --- |
| All cases | 17,228 | 100.0% | 13,183 | 100.0% | 4,045 | 100.0% |
| Nervous system disorders | 4,037 | 23.4% | 3,152 | 23.9% | 885 | 21.9% |
| Gastrointestinal disorders | 3,393 | 19.7% | 2,412 | 18.3% | 981 | 24.3% |
| General disorders and administration site conditions | 3,366 | 19.5% | 2,716 | 20.6% | 650 | 16.1% |
| Skin and subcutaneous tissue disorders | 2,736 | 15.9% | 2,264 | 17.2% | 472 | 11.7% |
| Psychiatric disorders | 2,045 | 11.9% | 1,680 | 12.7% | 365 | 9.0% |
| Investigations | 1,898 | 11.0% | 1,534 | 11.6% | 364 | 9.0% |
| Blood and lymphatic system disorders | 1,880 | 10.9% | 1,226 | 9.3% | 654 | 16.2% |
| Injury, poisoning and procedural complications | 1,823 | 10.6% | 1,365 | 10.4% | 458 | 11.3% |

## MedDRA Higher Level Terms (HLTs) and Preferred Terms (PTs)

**Table 4** Overview of MedDRA Preferred Terms (PT) summarized under MedDRA Higher Level Terms (HLTs). HLTs correspond to those reported on in manuscript Table 3

| **Higher Level Term (HLT)** | **Preferred Term (PT)** |
| --- | --- |
| Allergic conditions NEC | Hypersensitivity |
|  | Type II hypersensitivity |
|  | Type I hypersensitivity |
|  | Type IV hypersensitivity reaction |
|  | Type III immune complex mediated reaction |
|  | Erythema multiforme |
|  | Dermatitis allergic |
|  | Hypersensitivity pneumonitis |
| Anaemias NEC | Anaemia |
|  | Normochromic normocytic anaemia |
|  | Hypochromic anaemia |
|  | Normocytic anaemia |
|  | Normochromic anaemia |
| Anxiety symptoms | Anxiety |
|  | Agitation |
|  | Nervousness |
|  | Agitation postoperative |
| Asthenic conditions | Malaise |
|  | Asthenia |
|  | Fatigue |
|  | Lethargy |
| Coagulation and bleeding analyses | Activated partial thromboplastin time prolonged |
|  | Blood fibrinogen increased |
|  | Prothrombin time prolonged |
|  | International normalised ratio increased |
|  | Coagulation time prolonged |
|  | Coagulation test abnormal |
|  | Coagulation factor decreased |
|  | Prothrombin time abnormal |
|  | Prothrombin level decreased |
|  | International normalised ratio fluctuation |
|  | Prothrombin time ratio decreased |
|  | International normalised ratio abnormal |
|  | Prothrombin time shortened |
|  | Bleeding time prolonged |
| Coma states | Coma |
| Cortical dysfunction NEC | Confusional state |
|  | Aphasia |
|  | Apraxia |
|  | Agnosia |
| Deliria | Delirium |
| Diarrhoea (excl infective) | Diarrhoea |
| Disturbances in consciousness NEC | Depressed level of consciousness |
|  | Sedation |
|  | Loss of consciousness |
| Dyskinesias and movement disorders NEC | Tardive dyskinesia |
|  | Dyskinesia |
|  | Extrapyramidal disorder |
|  | Bradykinesia |
| Dyssomnias | Somnolence |
|  | Stupor |
|  | Poor quality sleep |
|  | Sleep apnoea syndrome |
|  | Hypersomnia |
| Febrile disorders | Pyrexia |
|  | Febrile infection |
|  | Hyperpyrexia |
|  | Febrile convulsion |
| Gait disturbances | Gait inability |
|  | Ataxia |
|  | Gait disturbance |
| Gastric ulcers and perforation | Gastric ulcer |
|  | Gastritis erosive |
|  | Gastric perforation |
|  | Gastric ulcer haemorrhage |
|  | Gastric ulcer perforation |
| Gastrointestinal atonic and hypomotility disorders NEC | Constipation |
|  | Oesophageal hypomotility |
|  | Gastrooesophageal reflux disease |
| General signs and symptoms NEC | Disease complication |
|  | Influenza like illness |
|  | Disease progression |
|  | Hot flush |
|  | General physical health deterioration |
|  | Mobility decreased |
|  | Concomitant disease aggravated |
|  | Crepitations |
|  | Condition aggravated |
|  | Balance disorder |
|  | Screaming |
|  | Crying |
|  | Pallor |
|  | Multiple organ dysfunction syndrome |
|  | Disease recurrence |
|  | Irritability |
|  | Swelling face |
|  | Foaming at mouth |
|  | Swelling |
|  | Dysstasia |
|  | Pre-existing condition improved |
|  | Illness |
|  | Ill-defined disorder |
| Haemorrhages NEC | Blood loss anaemia |
|  | Haemoptysis |
|  | Epistaxis |
|  | Injection site haematoma |
|  | Haematoma |
|  | Post procedural haematoma |
|  | Retroperitoneal haemorrhage |
|  | Post procedural haemorrhage |
|  | Haemorrhage |
|  | Wound haemorrhage |
|  | Procedural haemorrhage |
|  | Ulcer haemorrhage |
|  | Pericardial haemorrhage |
|  | Muscle haemorrhage |
|  | Bladder tamponade |
| Hallucinations (excl sleep-related) | Hallucinations, mixed |
|  | Hallucination, tactile |
|  | Hallucination, visual |
|  | Hallucination |
|  | Hallucination, auditory |
| Headaches NEC | Headache |
| Hepatobiliary function diagnostic procedures | Hepatic enzyme increased |
|  | Transaminases increased |
|  | Aspartate aminotransferase increased |
|  | Alanine aminotransferase increased |
|  | Gamma-glutamyltransferase increased |
|  | Blood bilirubin abnormal |
|  | Blood bilirubin increased |
|  | Liver function test increased |
|  | Liver function test abnormal |
|  | Hepatic enzyme abnormal |
|  | Gamma-glutamyltransferase |
| Hepatocellular damage and hepatitis NEC | Hepatocellular injury |
|  | Autoimmune hepatitis |
|  | Liver injury |
|  | Mixed liver injury |
|  | Cholestatic liver injury |
|  | Hepatic cytolysis |
|  | Hepatic necrosis |
|  | Hepatitis acute |
|  | Hepatitis |
|  | Hepatitis fulminant |
|  | Granulomatous liver disease |
| Interactions | Drug interaction |
|  | Potentiating drug interaction |
|  | Food interaction |
|  | Inhibitory drug interaction |
|  | Drug-disease interaction |
| Leukopenias NEC | Leukopenia |
|  | B-cell aplasia |
|  | Lymphopenia |
|  | Monocytopenia |
| Marrow depression and hypoplastic anaemias | Pancytopenia |
|  | Aplastic anaemia |
|  | Bone marrow failure |
|  | Bicytopenia |
|  | Myelosuppression |
|  | Febrile bone marrow aplasia |
|  | Pure white cell aplasia |
|  | Aplasia pure red cell |
| Mental disorders NEC | Altered state of consciousness |
|  | Mental disorder |
|  | Mental status changes |
| Muscle tone abnormal | Serotonin syndrome |
|  | Hypertonia |
|  | Hypotonia |
|  | Muscle rigidity |
|  | Neuroleptic malignant syndrome |
|  | Hypotonia neonatal |
| Nausea and vomiting symptoms | Vomiting |
|  | Nausea |
|  | Procedural nausea |
|  | Procedural vomiting |
|  | Retching |
|  | Regurgitation |
| Neurological signs and symptoms NEC | Dizziness |
|  | Respiratory depression |
|  | Restlessness |
|  | Clonus |
|  | Myoclonus |
|  | Disorientation |
|  | Personality change |
|  | Presyncope |
|  | Slow response to stimuli |
|  | Dizziness postural |
|  | Apnoeic attack |
|  | Unresponsive to stimuli |
|  | Neurological symptom |
|  | Head discomfort |
|  | Tongue movement disturbance |
| Neutropenias | Agranulocytosis |
|  | Neutropenic colitis |
|  | Febrile neutropenia |
|  | Neutropenia |
|  | Granulocytopenia |
| Non-site specific gastrointestinal haemorrhages | Upper gastrointestinal haemorrhage |
|  | Melaena |
|  | Gastrointestinal haemorrhage |
|  | Haematemesis |
|  | Haematochezia |
|  | Chronic gastrointestinal bleeding |
| Non-site specific injuries NEC | Fall |
|  | Wound |
|  | Foreign body |
|  | Accident |
|  | Road traffic accident |
|  | Wound infection |
| Off label uses | Off label use |
| Overdoses NEC | Intentional overdose |
|  | Overdose |
|  | Prescribed overdose |
| Poisoning and toxicity | Poisoning |
|  | Drug-induced liver injury |
|  | Toxicity to various agents |
|  | Toxic epidermal necrolysis |
|  | Stevens-Johnson syndrome |
|  | Accidental poisoning |
|  | Hepatotoxicity |
|  | Hepatitis toxic |
|  | Mucosal toxicity |
|  | Dermatitis contact |
|  | Carbon monoxide poisoning |
|  | Neurotoxicity |
|  | SJS-TEN overlap |
| Product administration errors and issues | Incorrect dosage administered |
|  | Product administration error |
|  | Wrong patient received product |
|  | Incorrect route of product administration |
|  | Incorrect dose administered |
|  | Accidental underdose |
|  | Accidental overdose |
|  | Inappropriate schedule of product administration |
|  | Contraindicated product administered |
| Rashes, eruptions and exanthems NEC | Rash maculo-papular |
|  | Rash |
|  | Rash macular |
|  | Rash erythematous |
|  | Rash papular |
|  | Rash pruritic |
|  | Rash vesicular |
|  | Rash morbilliform |
| Renal failure and impairment | Acute kidney injury |
|  | Renal failure |
|  | Anuria |
|  | Renal impairment |
|  | Chronic kidney disease |
|  | Oliguria |
|  | Haemolytic uraemic syndrome |
| Sepsis, bacteraemia, viraemia and fungaemia NEC | Neutropenic sepsis |
|  | Septic shock |
|  | Sepsis |
|  | Pulmonary sepsis |
|  | Urosepsis |
|  | Bacteraemia |
| Substance related and addictive disorders | Drug abuse |
|  | Drug withdrawal syndrome |
|  | Drug dependence |
|  | Withdrawal syndrome |
|  | Substance abuse |
|  | Drug withdrawal syndrome neonatal |
|  | Substance dependence |
|  | Alcohol problem |
| Therapeutic and nontherapeutic responses | Treatment failure |
|  | Drug ineffective |
|  | Drug ineffective for unapproved indication |
|  | Adverse event |
|  | Drug intolerance |
|  | Therapeutic product effect incomplete |
|  | Therapeutic response shortened |
|  | Drug tolerance decreased |
|  | Therapeutic product effect decreased |
|  | Therapeutic response increased |
|  | Multiple-drug resistance |
|  | Therapeutic response unexpected |
|  | Drug effective for unapproved indication |
|  | Drug tolerance |
|  | Therapeutic product effect increased |
|  | Similar reaction on previous exposure to drug |
|  | Therapeutic response decreased |
| Thrombocytopenias | Thrombocytopenia |
|  | Immune thrombocytopenia |

## Serious reactions and death

*Table 5 Sensitivity analysis: Seriousness and seriousness criteria of ICSRs of drugs used in pain therapies with an age stratification at 65 years. Note: A low ROR does not indicate a protective effect but suggests that the drug-event combination is not reported disproportionately in the database.*

|  | **Younger adults**  (18-64 years) | **Older adults** (65+ years) | **65+ years vs. younger** |
| --- | --- | --- | --- |
|  | Counts, No. (%) | Counts, No. (%) | ROR (95% CI) |
| **ICSRs** | 10129 (100.0) | 7099 (100.0) |  |
| **Serious** | | | |
| Yes | 5244 (51.8) | 4695 (66.1) | 1.8 (1.7-1.9)* |
| **Seriousness criteria** | | | |
| Hospitalization | 2833 (28.0) | 3134 (44.1) | 1.9 (1.6-1.8)* |
| Congenital | 0 (0.0) | 1 (0.0) | NA |
| Lifethreatening | 518 (5.1) | 301 (4.2) | 0.6 (0.5-0.7)* |
| Death | 230 (2.3) | 407 (5.7) | 2.1 (1.8-2.5) |
| Disabling | 170 (1.7) | 135 (1.9) | 0.9 (0.7-1.1) |
| Other | 2285 (22.6) | 1312 (18.5) | 0.5 (0.5-0.5)* |
| Missing | 27 (0.3) | 22 (0.3) | 0.9 (0.5-1.6) |
| * p < 0.05 | | | |

**Table 6** Top 3 substances and reactions most frequently in serious cases with death (seriousness criterion) in younger and older adults

| **Younger adults** (18-74 years) | | | | **Older adults** (75+ years) | | | |
| --- | --- | --- | --- | --- | --- | --- | --- |
| ICSR where reaction resulted in death | | 371 | 100.0% | ICSR where reaction resulted in death | | 266 | 100.0% |
| **1. Acetylsalicylic acid** | | **57** | **15.4%** | **1. Acetylsalicylic acid** | | **89** | **33.5%** |
|  | Central nervous system haemorrhages and cerebrovascular accidents | 47 | 12.7% |  | Central nervous system haemorrhages and cerebrovascular accidents | 60 | 22.6% |
|  | Haemorrhages NEC | 8 | 2.2% |  | Non-site specific gastrointestinal haemorrhages | 18 | 6.8% |
|  | Non-site specific gastrointestinal haemorrhages | 6 | 1.6% |  | Traumatic central nervous system haemorrhages | 12 | 4.5% |
| **2. Paracetamol** | | **41** | **11.1%** | **2. Metamizole** | | **25** | **9.4%** |
|  | Hepatic failure and associated disorders | 15 | 4.0% |  | Neutropenias /  Sepsis, bacteraemia, viraemia and fungaemia NEC | 12 12 | 4.5% 4.5% |
|  | Poisoning and toxicity | 12 | 3.2% |  | Marrow depression and hypoplastic anaemias | 11 | 4.1% |
|  | Hepatocellular damage and hepatitis NEC | 6 | 1.6% |  | Lower respiratory tract infections NEC | 6 | 2.3% |
| **3. Morphine** | | **24** | **6.5%** | **3. Paracetamol** | | **25** | **9.4%** |
|  | General signs and symptoms NEC | 6 | 1.6% |  | Marrow depression and hypoplastic anaemias | 5 | 1.9% |
|  | Poisoning and toxicity | 5 | 1.3% |  | Hepatocellular damage and hepatitis NEC /  Renal failure and impairment | 4  4 | 1.5%  1.5% |
|  | Overdoses NEC /  Substance related and addictive disorders | 4 4 | 1.1% 1.1% |  | Cholestasis and jaundice | 3 | 1.1% |
